# Proteomics-Based Identification of Salivary Changes in Patients with Burning Mouth Syndrome

**DOI:** 10.3390/biology10050392

**Published:** 2021-05-01

**Authors:** Candela Castillo-Felipe, Lorena Franco-Martínez, Asta Tvarijonaviciute, Pia Lopez-Jornet, Elsa Lamy

**Affiliations:** 1 Faculty of Medicine and Odontology, Hospital Morales Meseguer, Clínica Odontológica, 30008 Murcia, Spain; candela.castillo@um.es; 2 Interdisciplinary Laboratory of Clinical Analysis (Interlab-UMU), Regional Campus of International Excellence “Campus Mare Nostrum”, University of Murcia, 30100 Murcia, Spain; lorena.franco2@um.es; 3 Faculty of Medicine and Odontology, Biomedical Research Institute (IMIB-Arrixaca) Hospital Morales Meseguer, Clínica Odontológica, 30008 Murcia, Spain; majornet@um.es; 4 Mediterranean Institute for Agriculture, Environment and Development (MED), Institute for Advanced Studies and Research, University of Evora, 7006-554 Evora, Portugal; ecsl@uevora.pt

**Keywords:** burning mouth syndrome, saliva, proteomics

## Abstract

**Simple Summary:**

Burning mouth syndrome (BMS) is a chronic oral condition characterized by an intraoral burning sensation, taste alterations, and dry mouth sensations. The disease affects 0.7–15% of the general population, being most common in post-menopausal women. Although BMS is related to anxiety and/or depression and sleep disturbances, its etiology as well as its diagnosis remain unclear. Therefore, the present study aimed to contribute to the knowledge about this syndrome and to look for objective diagnostic tools. Therefore, whole saliva proteomes of patients suffering from BMS were compared to those of healthy persons. The results of this study manifest alterations in salivary proteins related to stress, immune system, and inflammation and, therefore, suggest implication of these pathways in BMS development. Moreover, biomarkers related to stress, immune system, and inflammation, such as salivary amyloid A, immunoglobulins, or leukocyte elastase inhibitors, among others, could contribute to BMC management, although further research is needed to confirm these suppositions.

**Abstract:**

Burning mouth syndrome (BMS) is a chronic oral condition characterized by an intraoral burning sensation, taste alterations, and dry mouth sensations. Although a number of factors have been closely related to the appearance of the symptoms, including anxiety, depression, and sleep disturbances, the etiology of BMS remains unclear. Furthermore, currently no objective diagnostic tools exist, making its diagnosis challenging. Therefore, to contribute to the knowledge about BMS etiology and look for objective tools for its diagnosis, the present study was conducted. Thus, the aim of this study was to analyze the proteomic profile of the resting whole saliva of patients with BMS and age and sex-matched controls using two-dimensional electrophoresis. The results showed evidence of changes in saliva at the level of proteins related to important pathways such as stress (sAA), immune system (Ig), and inflammation (leukocyte elastase inhibitor). While some of our findings have been previously described others, such as the deregulation of the coiled-coin domain containing protein 25 in BMS, are presented here for the first time to our knowledge. Thus, saliva provides us with relevant information about BMS pathophysiology and could be considered a suitable biofluid for its study and/or diagnosis.

## 1. Introduction

The Third Edition of the International Classification of Headache Disorders (2018) has defined burning mouth syndrome (BMS) as an “intraoral burning sensation or dysesthesia manifesting daily for more than two hours during a period of over three months, with no clinically manifest causal lesions” [1]. The literature also contemplates other terms describing this disorder, such as glossodynia or stomatopyrosis, referring mainly to a burning sensation of the oral mucosa and not to the global symptoms that conform the syndrome. The term “burning mouth syndrome”, therefore, is considered to be the most appropriate [2]. The etiology of BMS is unclear, although a number of factors have been closely related to the appearance of the symptoms, such as anxiety and/or depression, and sleep disturbances [3,4]. Taste alterations (dysgeusia) and dry mouth (xerostomia) also have been described [5,6]. Due to the indistinctness of the pathology, the diagnosis also is complicated and often controversial and is basically dependent upon the clinical manifestations [7,8]. Furthermore, in approximately 3% of patients, burning symptoms spontaneously disappear five years after their appearance [9], making this disease even more difficult to understand and diagnose.

Saliva has gained special importance in recent years as a diagnostic fluid, since it is easy to obtain, noninvasive, and economical. More than 3000 proteins have been identified in saliva, constituting potential biomarkers of different pathologies [10]. Differences have been found in the saliva of patients with BMS with respect to healthy controls in biomarkers of the immune system (Immunoglobulin A, macrophage inflammatory protein-4), adrenergic system (salivary α-amylase), and oxidative stress (uric acid and plasma iron reduction capacity), among others [5,6,11]. Furthermore, associations between salivary biomarkers and clinical variables such as pain and psychological disorders were detected [5,6,11].

Proteomic studies relate their presence with different physiological and metabolic states, and help in clarifying the role of each of those proteins. This is very useful, since proteins that are either over- or under-expressed in a given disease process may serve as biomarkers of the disease [12]. Proteomics, therefore, is a very important tool that can define a specific altered protein profile for each disease condition, thus contributing to the understanding of the underlying pathophysiology and serving as a diagnostic and monitoring tool, as well as opening new treatment perspectives [12]. Salivary proteomic profiles were studied in a number of oral disorders such as oral squamous cell carcinoma (OSCC), Sjögren’s syndrome, periodontitis, and BMS [13]. Proteomic studies performed in BMS were conducted using different methodologies, isobaric tags for relative and absolute quantitation labelling and liquid chromatography-tandem mass spectrometry [14], liquid chromatography coupled with electrospray-ionization mass spectrometry [15], and two dimensional gel electrophoresis (2-DE) after depletion of 21 high abundance proteins [16]. Concerning all cases, salivary proteoms of patients with BMS and of controls were performed.

Considering the need for objective diagnostic tests that can contribute to clarifying the causes of the symptoms in patients with BMS, the present study was conducted. The aim of this study was to analyze the proteomic profile of the resting whole saliva of patients with BMS, as well as that of age and sex-matched controls, using two-dimensional electrophoresis in a simple and noninvasive manner. This gel-based method was chosen since (1) it allows the visualization of different forms (e.g., phosphorylation, glycosylation, etc.) of the most abundant salivary proteins; (2) the depletion of the most abundant proteins could alter the profile of the less abundant proteins, therefore untreated whole saliva samples were used in this study; and (3) it allows the identification of proteins that are present in relatively high concentrations that would allow their subsequent determination with specific methods and, thus, be used in a clinical setting.

## 2. Materials and Methods

### 2.1. Study Design and Subjects

The present cross-sectional study was conducted in the Unit of Oral Medicine (University of Murcia, Spain) and in the Mediterranean Institute for Agriculture, Environment and Development (MED, University of Évora, Portugal) between the months of September 2019 and September 2020. The study complied with the Declaration of Helsinki and was approved by the Ethics Committee of the University of Murcia (Ref.: 2203/2018). All patients gave informed consent to participation. The clinical data and salivary samples were collected in the University of Murcia, and the proteomics analysis was performed in the laboratories of the University of Évora.

### 2.2. Sample Description

A total of 11 patients (6 females) with the age range between 42 and 83 years (mean, 54 years), were included in the study. The patients were diagnosed with BMS according to the criteria of the International Classification of Headache Disorders (2018) (1): “intraoral burning sensation or dysesthesia manifesting daily for more than two hours during a period of over three months, with no clinically manifest causal lesions”.

The control group in turn consisted of 11 age and sex-matched healthy subjects (6 females) with ages between 40 and 88 years (mean, 54 years).

All participants underwent oral exploration by the same expert in oral medicine (CCF), with the evaluation of oral and dental health. Regarding all cases, exclusion criteria included the presence of poor oral hygiene, caries, periodontitis, smoking, as well as pregnant women and individuals subjected to chemotherapy/radiotherapy, or those with any oral condition capable of accounting for the symptoms were excluded.

Six patients with BMS and 3 controls were users of dentures (Chi-square, *p* = 0.193).

### 2.3. Saliva Collection

Resting whole saliva was collected using the drainage technique, with a collection time of 5 min. To avoid possible contamination from other sources, the patients were instructed to rinse the mouth thoroughly before saliva sample collection. The subjects were required to avoid heavy physical exercise and abstain from food and drink intake during one hour before sampling. The samples were collected at about the same time in all subjects (8:00–11:00 a.m.). Immediately after collection, samples were placed in coolers and transported to the laboratory, where saliva was vortexed and centrifuged (3000× *g* for 10 min at 4 °C), and the supernatant was transferred into polypropylene tubes and stored at −80 °C until analysis.

### 2.4. Two-Dimensional Electrophoresis, Computational Image, and Mass Tandem Spectrometry

Protein content was determined using the Bradford Determination [17], and 150 milligrams of proteins from each saliva sample were employed. The quantitative proteomic analysis consisted of two-dimensional polyacrylamide gels followed by mass spectrometry (MS), which were run in duplicate, as described elsewhere [18]. Briefly, for each salivary sample isoelectric focusing was conducted in 3-11 pH non-linear (NL) immobilized pH gradient (IPG) strips of 7 cm length (GE Healthcare Life Sciences); while the second dimension was performed after reduction and alkylation of the IPG strips. Sodium-dodecyl sulphate-polyacrylamide electrophoresis (SDS-PAGE) protein separation was performed in homemade 14% polyacrylamide gels. Gels were stained using Coomassie Brilliant Blue R-250 (2% CBB, 40% methanol, 10% acetic acid), and destained in 10% acetic acid, using a protocol compatible with MS analysis.

The 2-DE gels were digitalized and analyzed using ImageMaster v7.0 software (GE Healthcare Life Sciences, Piscataway, NJ, USA). Spot detection was made automatically and edited manually. The match analysis included manual alignment by defining landmarks distributed over each entire image that clearly represented the same protein form, after which automatic matching was done and posteriorly revised for confirmation. To identify those protein spots which differed significantly in abundance between the control and test gels, the software for analysis (Metaboanalyst, https://www.metaboanalyst.ca/, accessed on 1 June 2020) was used.

Mass spectrometry was performed as described elsewhere [19]. Briefly, those spots that showed differences of statistical relevance between the two groups were manually excised, washed, destained, reduced, and alkylated [20,21]. After tryptic digestion for 8 h at 37 °C (Trypsin Gold, Mass Spectrometry Grade, Pro-mega, Madison, WI, USA), peptides were extracted using three portions of 30 μL of 5% trifluoro acidic acid in 50% aqueous acetonitrile and dried in a vacuum concentrator (Eppendorf, Hamburg, Germany). Peptides were then separated and analyzed using Ekspert nano LC 425 (Sciex) coupled to a high resolution quadrupole time-of-flight mass spectrometer (Triple TOF 6600, Sciex). The database for protein identification was downloaded from the UniProt database (www.uniprot.org, accessed on 1 June 2020).

### 2.5. Statistical and Bioinformatics Analysis

Regarding analysis of gel spots, as data did not follow Gaussian distribution, data were log-transformed and a Student’s *T*-test was performed to compare each spot abundance between the two groups, while multivariate analyses were performed to consider potential interactions among protein spots in group comparisons. PLS-DA (partial Least Square-Discriminant Analysis) was performed for this, and VIP (Variable Importance in Projection) scores higher or equal to 2.0 were considered to discern the spots contributing most to group separation. All *p*-values < 0.05 were considered to be significantly differentially expressed.

Genes encoding the differentially abundant proteins between the BMS and healthy groups were used to determine the Gene Ontology (GO) terms over-represented in BMS using the Protein Analysis Through Evolutionary Relationships (PANTHER) classification tool (http://www.pantherdb.org/, accessed on 1 June 2020).

## 3. Results

No statistically significant differences were detected between patients with burning mouth syndrome and the controls in terms of salivary total protein concentration (patients, 569.9 ± 254.8 µg/mL vs. controls, 537.3 ± 196.0 µg/mL; *p* = 0.745) and salivary flow rate (BMS patients, 3.7 ± 1.6 mL vs. controls, 2.3 ± 1.6 mL; *p* = 0.132).

**Figure 1:**
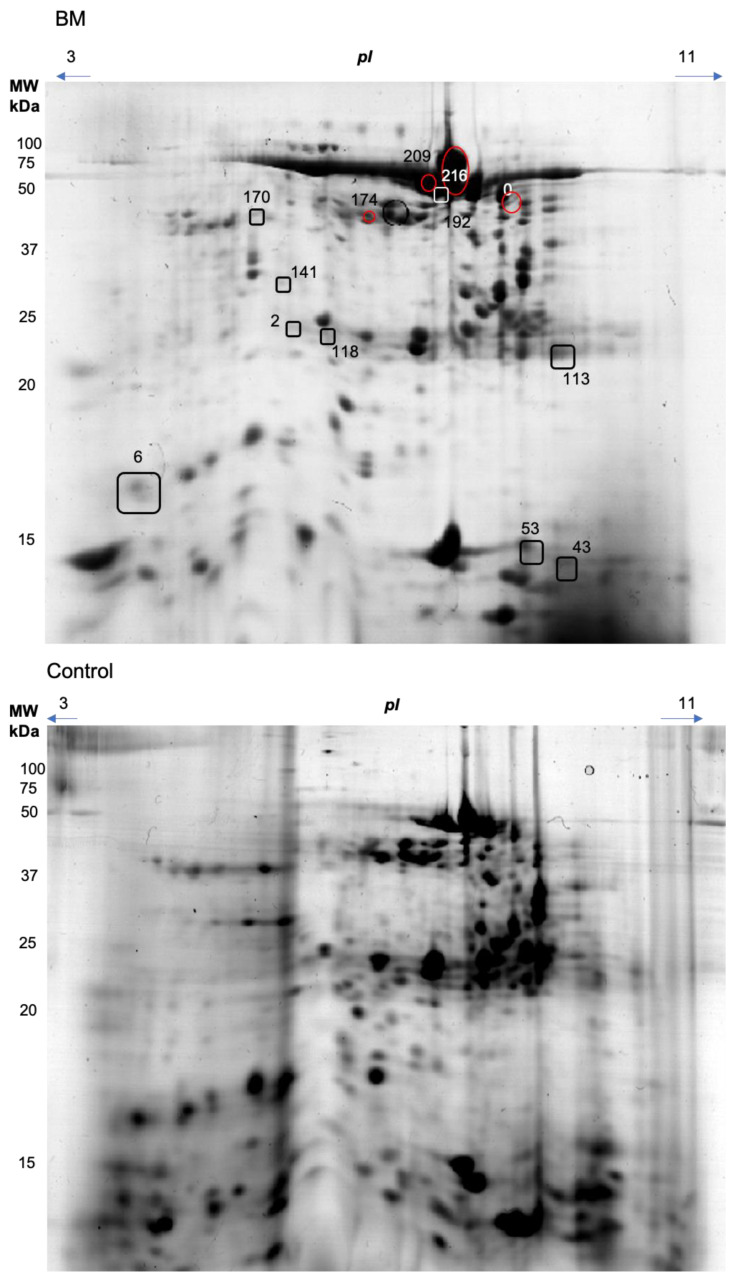
Representative two-dimensional gel electrophoresis of salivary proteins of patient with BMS (upper) and healthy control (lower). Proteins with statistically significantly different abundance between groups were circled in red (higher in BMS) or squared (higher in healthy control group).

Representative gel images are shown in Figure 1. The 2D image analysis revealed a total of 141 spots that were matched between the two groups. Considering those, nine spots (2, 43, 58, 118, 141, 170, 174, 192, 209) were observed to present a different relative abundance between the healthy and BMC groups (*p* < 0.05), when univariate analysis was performed and, therefore, were selected for identification by mass spectrometry (Table 1).

**Figure 2:**
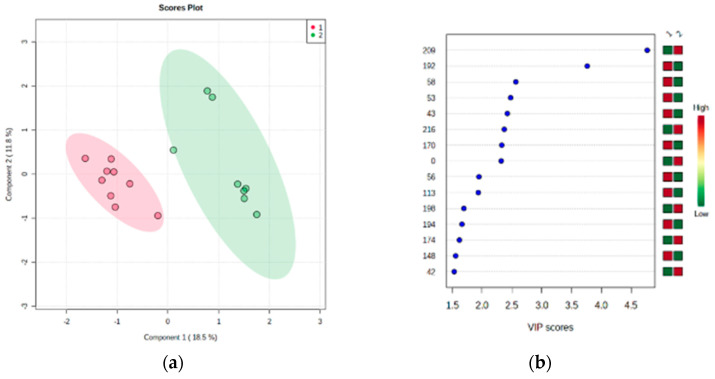
(**a**) Two-dimensional representation of components from Partial Least Square-Discriminant Analysis (PLS-DA), showing clear separation between healthy (1) and burning mouth syndrome (2) groups. (**b**) VIP (Variable Importance in Projection) describing the spots contributing most to group separation.

Partial Least Square—Discriminant Analysis revealed that groups of healthy individuals can be distinguished from BMS patients (Figure 2). The BMS groups nested distantly from healthy groups in component 1, which explained 18.5% of the total variance. Ten spots presented VIP scores greater or equal to 2.0 (0, 43, 53, 56, 58, 113, 116, 170, 192, 209), as identified by MS since they were among those that contributed most to group separation (Table 1).

A total of ten proteins were identified as composing the nine statistically significant modulated spots, which represented differences between the two groups. These were albumin, alpha-amylase 1, coiled-coil domain-containing protein 25, cystatin-S, cystatin-SN, dermcidin, immunoglobulin heavy constant alpha 1, immunoglobulin kappa constant, leukocyte elastase, prolactin-inducible protein, and protein shroom 3. Seven spots, composed by the all above mentioned proteins, were down-regulated in BMS, with a fold change (Group1/Group2) ranging from 4.92- to 15.3-folds lower in abundance.

To contrast, four spots (0, 174, 209, and 216) containing alpha-amylase 1, immunoglobulin heavy constant alpha 1, and dermcidin or coiled-coil domain-containing protein 25 were most represented in the BMS group, with an up to 14-folds higher abundance.

**Figure 3:**
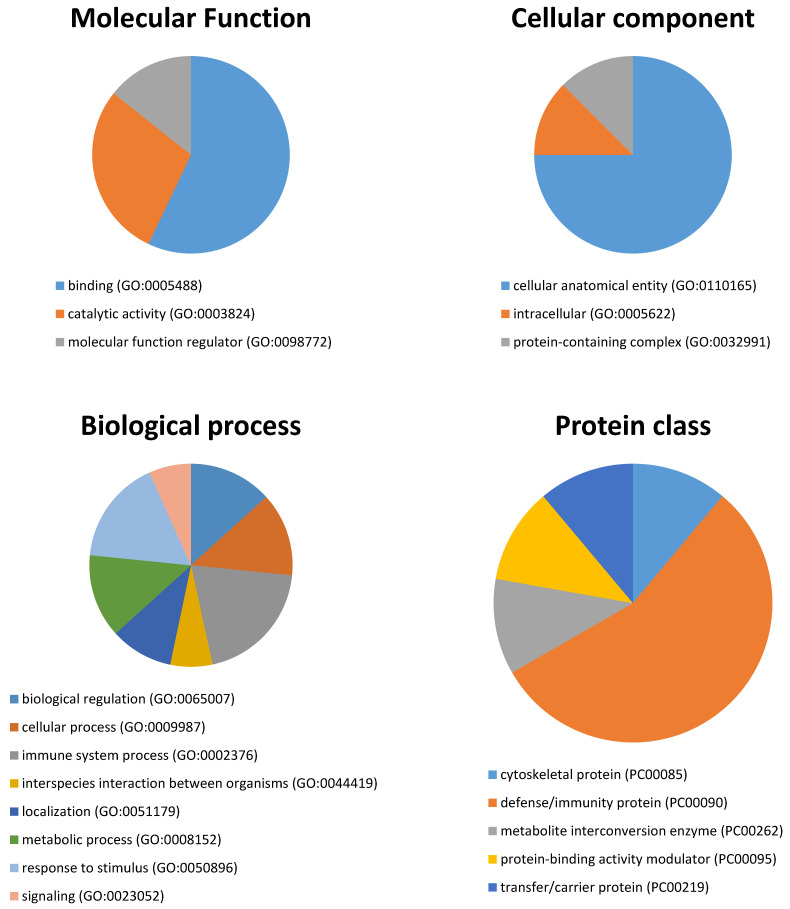
Pie charts showing the molecular function, cellular component, biological process, and protein class between BMS and healthy control groups based on the PANTHER classification system (http://www.pantherdb.org, accessed on 1 June 2020).

The fourteen unique genes identified in the spots differing in expression levels between the two groups or contributing to group separation were used for subsequent bioinformatic analyses in terms of functional clusters, according to the PANTHER classification system, as shown in Figure 3. The proteins were distributed within three different molecular functions: binding (GO:0005488) (57.1%), catalytic activity (GO:0003824) (28.6%), and molecular function regulator (GO:0098772) (14.3%). Cellular anatomical entity (GO:0110165) was the main cellular component (75%), followed by intracellular (GO:0005622), and protein-containing complex (GO:0032991) (12.5% each). They participate in eight molecular processes, namely the immune system process (GO:0002376) (20%), response to stimulus (GO:0050896) (16.7%), metabolic process (GO:0008152), cellular process (GO:0009987), and biological regulation (GO:0065007) (13.3% each), localization (GO:0051179) (10%), interspecies interaction between organisms (GO:0044419), and signaling (GO:0023052) (6.7% each). Last, five protein classes were identified, being the defense/immunity protein (PC00090) (the most represented at 55.6%), followed by the cytoskeletal protein (PC00085), the metabolite interconversion enzyme (PC00262), the protein-binding activity modulator (PC00095), and the transfer/carrier protein (PC00219) at 11.1% each.

## 4. Discussion

During the present study, 13 spots differentially modulated between patients with BMS and healthy controls were observed. Fourteen different unique genes were identified from these spots, with most of them involved in the immune system, cellular, and metabolic processes. Some of the proteins identified in this study, including albumin, immunoglobulins, dermcidin, and alpha-amylase were previously reported to be modulated in BMS, validating our findings [11,14,15,16], while others, such as coiled-coil domain-containing protein 25, were related to BMS herein for the first time. Furthermore, our results were in accordance with the previous data analyzing salivary proteomes in healthy individuals and BMS patients using different proteomic approaches [14,15,16], although some divergences are present possibly due to the different populations and study designs used. Ji et al. [14] identified 1130 proteins, using isobaric tags for relative and absolute quantitation labeling and liquid chromatography-tandem mass spectrometry, having 39 differentially modulated between the two groups and three proteins (alpha-enolase, interleukin-18, kallikrein-13) confirmed by ELISA. Cabras et al. [15] investigated different proteins by liquid chromatography coupled to electrospray-ionization mass spectrometry in different saliva samples and only detected alterations in cystatin SN protein, which was up-regulated in patients with BMS. These differences, where decreases in S-type cystatin spots were observed, may be executed in different proteomic approaches relative to our study. Cabras et al. [15] did not separate the different forms of cystatin SN protein, whereas two-dimensional electrophoresis (2DE) does this and allows us to observe the differences in each of them. Last, Krief et al. [16] performed qualitative and quantitative two dimensional gel electrophoresis (2-DE); however, and in contrast to this study, they previously performed depletion of 21 high abundance proteins. Although protein depletion increases sensitivity for low-abundant proteins, it can affect the levels/ratios of non-targeted proteins for depletion and cannot reveal changes in depleted ones such as alpha-amylase or immunoglobulins that are known to change in BMS [11]. Nevertheless, Krief et al. [16] identified 100 BMS-specific proteins and 158 proteins up-regulated in BMS, more than threefold in comparison with healthy controls. Overall, despite these differences, all studies outline alterations in immune system function and, therefore, immune system biomarkers could be employed for improving BMS diagnosis and treatment.

Only one of the differentially modulated spots was composed of a single protein (leukocyte elastase inhibitor (SERPINB1)), which was down-regulated in BMS. This protein plays a major role in the regulation of the innate immune response, cellular homeostasis and inflammation, and in protecting cells from proteases. This result is in contrast to the data reported by Krief et al. [16], who found higher SERPINB1 in patients with BMS in comparison to healthy controls. It also is important to notice that, although some discrepancies exist, SERPINB1 in saliva was related to periodontal inflammation [22,23]. Thus, the contradictory results regarding SERPINB1 obtained in the present and previous [16] studies could be attributed to the different proteomic approaches employed and/or different populations involved in terms of oral health status and BMS evolution. Therefore, further studies evaluating SERPINB1 in patients with BMS, with and without periodontal inflammation, should be performed to clarify these results.

Salivary alpha-amylase (sAA) was identified in six spots analyzed by MS. SAA is a sensitive biomarker of stress [24]. A number of studies described increased total salivary alpha-amylase levels in patients with BMS in comparison to healthy controls, possibly related to physical pain and stress due to disease [6,25]. However, two spots that contained sAA did not differ statistically between study groups; two were of higher and two of lower abundance in BMS. These different abundances in relation to controls of the spots containing sAA observed in the present study could be explained by two facts: (1) spots contain more than one protein/isoform of protein that could be differentially affected by the presence of BMS, giving a different total abundancy in relation to controls; and (2) sAA is present in more than 140 gel spots due to post-translationally modified forms and isoforms that could be altered differently in different situations [26]. Briefly, it is possible that IgA contributes to the lower abundance of spot 39 rather than amylase and, since spot 29 has a lower apparent molecular mass than the usual forms of sAA, it probably can result in proteolysis. However, future studies are needed to assess the response of different sAA isoforms to BMS.

MS analyses identified cystatin SN in two spots under-regulated in BMS. Although the discrepancy could be explained by the identification of other proteins in the same spots and by the differential analyses used, this finding was in discordance with Cabras, T. et al. [15] who found that cystatin was overexpressed in unstimulated saliva from BMS patients, suggesting a relationship between cystatin SN and inflammation. Another possible explanation for this discrepancy could be that Cabras et al. did not perform a clinical evaluation to verify whether patients were affected by periodontitis or gingivitis, which are known to affect the levels of salivary cystatins [27]; while, in the present study, patients were excluded if presenting these conditions. Changes in cystatin SN in BMS patients could reflect a defensive reaction against an on-going inflammation, which may be one of the subjacent causes for BMS.

Immunoglobulins (alpha and kappa) were identified in deregulated spots and contributed to group differentiation. Immunoglobulins are key proteins against pathogens, although changes in salivary IgA also have been related to stress [28] and, in agreement with our results, differences in immunoglobulins were previously observed in patients with BMS in comparison to healthy individuals [6,11,25,29].

Albumin also was present in two spots of lower abundance in BMS, although other proteins also were detected in these spots. This result was in agreement with previous studies that reported higher levels of albumin and immunoglobulins in saliva in BMS patients [6]. The authors reported the serum-borne (and not salivary gland) origin of these proteins that leaked to saliva due the microscopic atrophy of the oral mucosa, has been reported in 70% of BMS patients [30].

To the best of our knowledge, coiled-coil domain-containing protein 25 (CCDC25) has been described in relation to BMS in this study for the first time. This protein is highly conserved among mammals and its expression is almost ubiquitous in human tissues; however, its function is still unclear [31]. This protein has been proposed recently as a serum biomarker of chronic cholangitis and cholangiocarcinoma [31]. However, there is no data about CCDC25 and saliva or BMS and, therefore, further studies should be performed to clarify its possible relation.

Our study offers a number of novel data. Basically, it seeks to transfer the information obtained from advanced proteomic techniques to clinical practice. These new technologies are known to generate panels of potential biomarkers that must be validated and should be traceable with the use of less costly analytical techniques to allow their transfer to daily practice. Consequently, clinical trials are indicated for confirmation that the salivary protein profiles differ between groups. Our study also has the advantage of complementing previous studies, using a different proteomic approach consisting of 2-dimensional gel electrophoresis in non-depleted salivary samples. Although gel-based proteomics is more time consuming, both in terms of laboratory and image analysis, allowing only the analysis of proteins that enter the gels (as such, excluding high or low molecular mass proteins, as well as proteins with low solubility and extreme isoelectric points), this technique allows the visualization of the different forms of the same protein. Regarding saliva where abundant proteins, such as amylase and cystatins, among others, exist in different isoforms, it allows us to compare them and to search for involvement at an individual level. However, the proteomic methodologies are not limitation-free. The macrophage inflammatory protein-4 (MIP4), for instance, was not identified in this study, while it was observed to have statistically significant higher concentrations in the saliva of patients with BMS compared to controls when a specific immunologic method was used [11]. Therefore, the use of proteomic analysis should be considered as complementary and not singular for new disease biomarker identification. Regarding the limitations of our study, mention must be made of the small sample size and the case-control design involved. Further large-scale trials are needed to confirm our findings and gain further knowledge of the possible associations between these proteins and BMS.

## 5. Conclusions

The proteomic study performed evidenced changes in saliva that affect proteins related to important pathways such as stress (sAA), the immune system (Ig), and inflammation (leukocyte elastase inhibitor). While some of our findings have been previously described others, such as the deregulation of the coiled-coin domain containing protein 25 in BMS, have been described here for the first time. Thus, saliva provides us with relevant information about BMS pathophysiology and could be considered as a suitable biofluid for its study and/or diagnosis.

## Figures and Tables

**Table 1:** Mass spectrometry results on identified spots. Abundances are expressed as median (25–75 percentile).

Spot	Spectra	Peptides	Score	Coverage	Intensity	Protein mw	Accession Number	Entry Name	Control	Burning Mouth Syndrome	*p*
58	5	4	34.68	4.9	2.22 × 10^4^	69,365.5	P02768	Albumin	0.684 (0426–0.684)	0.086 (0.029–0.421)	0.001
	5	3	31.59	17.8	6.65 × 10^4^	16,572.2	P12273	Prolactin-inducible protein			
	5	3	30.68	25.4	2.44 × 10^4^	11,283.7	P81605	Dermcidin			
	2	1	14.54	4.3	2.43 × 10^4^	24,478.4	Q86WR0	Coiled-coil domain-containing protein 25			
118	12	7	84.61	12.9	1.35 × 10^5^	57,766.8	P04745	Alpha-amylase 1	0.207 (0.074–0.207)	0.005 (0.005–0.056)	0.023
	6	3	32.42	47.6	6.03 × 10^4^	11,764.8	P01834	Immunoglobulin kappa constant			
192	81	24	362.45	51.6	3.20 × 10^6^	57,766.8	P04745	Alpha-amylase 1	1.038 (0.595–1.285)	0.121 (0.326–1.090)	0.002
	5	3	36.75	8.7	4.16 × 10^4^	37,654	P01876	Immunoglobulin heavy constant alpha 1			
	2	1	11.96	4.3	2.33 × 10^4^	24,478.4	Q86WR0	Coiled-coil domain-containing protein 25			
2	4	3	28.1	3.4	2.45 × 10^4^	69,365.5	P02768	Albumin	0.118 (0.052–0.198)	0.007 (0.007–0.051)	0.004
	1	1	15.97	4.3	9.20 × 10^3^	24,478.4	Q86WR0	Coiled-coil domain-containing protein 25			
141	7	4	49.52	24.5	5.03 × 10^4^	11,283.7	P81605	Dermcidin	0.116 (0.070–0.132)	0.007 (0.007–0.028)	0.0001
	1	1	11.92	4.3	2.11 × 10^4^	24,478.4	Q86WR0	Coiled-coil domain-containing protein 25			
	2	1	10.93	0.4	3.13 × 10^4^	21,6853.7	Q8TF72	Protein Shroom3			
170	9	9	97.49	24.2	5.68 × 10^4^	42,741	P30740	Leukocyte elastase inhibitor	0.476 (0.157–0.600)	0.012 (0.012–0.047)	0.0004
43	7	5	49.82	34.5	6.48 × 10^4^	11,283.7	P81605	Dermcidin	0.330 (0.266–0.658)	0.013 (0.013–0.053)	0.0004
	6	3	34.96	33.3	9.31 × 10^4^	16,387.4	P01037	Cystatin-SN			
209	19	13	147.03	25.8	2.05 × 10^5^	57,766.8	P04745	Alpha-amylase 1	0.049 (0.049–0.147)	1.586 (0.841–3.629)	0.001
	4	3	36.45	7.9	2.69 × 10^4^	37,654	P01876	Immunoglobulin heavy constant alpha 1			
	2	2	16.14	15.4	1.08 × 10^4^	11,283.7	P81605	Dermcidin			
174	8	6	79.02	11.3	9.81 × 10^4^	57,766.8	P04745	Alpha-amylase 1	0.011 (0.011–0.147)	0.206 (0.159–0.427)	0.00005
	7	4	56.48	9.9	7.86 × 10^4^	37,654	P01876	Immunoglobulin heavy constant alpha 1			
	2	1	12.29	4.3	1.16 × 10^4^	24,478.4	Q86WR0	Coiled-coil domain-containing protein 25			
0	56	22	298.52	51.2	2.01 × 10^6^	57,766.8	P04745	Alpha-amylase 1	0.027 (0.027–0.027)	0.382 (0.142–0.539)	0.049
113	12	4	55.29	60.7	2.95 × 10^5^	11,764.8	P01834	Immunoglobulin kappa constant	0.512 (0.024–0.890)	0.024 (0.024–0.094)	0.030
	2	2	22.19	13.4	1.74 × 10^4^	13,147.6	A0A0C4DH55	Immunoglobulin kappa variable 3D-7			
	2	2	18.76	13.9	1.32 × 10^4^	12,625	A0A0A0MRZ8	Immunoglobulin kappa variable 3D-11			
	3	2	15.57	13.7	2.04 × 10^4^	12,556.9	P01619	Immunoglobulin kappa variable 3-20			
216	143	27	468.85	66.9	2.37 × 10^7^	57,766.8	P04745	Alpha-amylase 1	0.013 (0.013–0.013)	0.105 (0.025–0.146)	0.016
53	27	7	91.17	63.1	1.16 × 10^6^	16,387.4	P01037	Cystatin-SN	1.914 (1.351–3.299)	0.709 (0.211–1.823)	0.035
	18	4	50.6	34	1.05 × 10^6^	16,214.1	P01036	Cystatin-S			

## Data Availability

Data is available under reasonable request.
